# Endocrine and Metabolic Signaling in Retroperitoneal White Adipose Tissue Remodeling during Cold Acclimation

**DOI:** 10.1155/2013/937572

**Published:** 2013-04-24

**Authors:** Aleksandra Jankovic, Aleksandra Korac, Biljana Buzadzic, Vesna Otasevic, Ana Stancic, Milica Vucetic, Milica Markelic, Ksenija Velickovic, Igor Golic, Bato Korac

**Affiliations:** ^1^Department of Physiology, Institute for Biological Research “Sinisa Stankovic”, University of Belgrade, 11060 Belgrade, Serbia; ^2^Faculty of Biology, Center for Electron Microscopy, University of Belgrade, 11000 Belgrade, Serbia

## Abstract

The expression profiles of adiponectin, resistin, 5′-AMP-activated protein kinase **α** (AMPK**α**), hypoxia-inducible factor-1**α** (HIF-1**α**), and key enzymes of glucose and fatty acid metabolism and oxidative phosphorylation in rat retroperitoneal white adipose tissue (RpWAT) during 45-day cold acclimation were examined. After transient suppression on day 1, adiponectin protein level increased following sustained cold exposure. In parallel, on day 1, the protein level of HIF-1**α** was strongly induced and AMPK**α** suppressed, while afterwards the reverse was seen. What is more, after an initial decrease on day 1, a sequential increase in pyruvate dehydrogenase, acyl-CoA dehydrogenase, cytochrome *c* oxidase, and ATP synthase and a decrease in acetyl-CoA carboxylase (from day 3) were observed. Similar to adiponectin, protein level of resistin showed a biphasic profile: it increased after days 1, 3, and 7 and decreased below the control after 21 days of cold-acclimation. In summary, the data suggest that adiponectin and resistin are important integrators of RpWAT metabolic response and roles it plays during cold acclimation. It seems that AMPK**α** mediate adiponectin effects on metabolic remodeling RpWAT during cold acclimation.

## 1. Introduction

Acclimation to low temperature induces profound shifts in energy expenditure aimed at maintaining a stable body temperature [1]. Many aspects of adaptive metabolic changes in muscles, brown adipose tissue, and liver as the sites of thermoregulatory thermogenesis, that is, tissues with the highest oxygen consumption, have been the subject of considerable research [2–7]. By contrast, the data related to white adipose tissue (WAT) during cold acclimation are mainly focused on the metabolic pathways of the synthesis and breakdown of triglycerides, as ways of controlling fatty acids release, for example, availability for other tissues [8, 9], as well as on the morphological plasticity—the appearance of multilocular, brown fat-like cells [10, 11].

It is known that the response of white fat tissue in the conditions of increased whole body energy demands involves complex recruitment in WAT at the metabolic and endocrine levels. More recent developments in adipose tissue research have shown that WAT integrates metabolic signals and rapidly responds to changing environments by synthesizing endocrine factors adipokines. These factors initiate powerful feedback actions at the systemic level involved in the regulation of food intake and energy expenditure, that is, metabolic homeostasis. What is more, these factors exert local paracrine and autocrine actions, strongly affecting the metabolic response of WAT and its metabolic role in different metabolic states [12].

The most important energy-metabolism related adipokines are leptin, adiponectin, and resistin. The effects of cold exposure on leptin synthesis in WAT and the role it plays in cold adaptation are well known [13–18], while data on the expression changes and functions of adiponectin and resistin are largely unknown. Adiponectin regulates glucose uptake, lipogenesis, lipolysis, and fatty acid oxidation in various tissues including WAT [19–22], and resistin influences gluconeogenesis and reesterification in liver and WAT, respectively [23], metabolic processes that are strongly affected during cold acclimation [2, 9, 24–26].

5′-AMP-activated protein kinase (AMPK) and hypoxia-inducible factor-1 (HIF-1) are the prime sensors of acute cellular energetic stress that trigger different metabolic strategies in energy production, thus directing long-term cellular metabolism. On the other hand, both of these metabolic regulators are recognized as important mediators of energy-related adipokine synthesis and/or action [27–29]. The physiological roles of HIF-1 and AMPK interplay in white fat metabolism and the expression of adiponectin and especially in relation to resistin are still unknown.

In this study, we aimed to examine the endocrine and metabolic signaling related to WAT energy metabolism during cold acclimation in rats. Therefore, we focused on the expression profiles of adiponectin and resistin, AMPK*α*, and HIF-1*α*, and their potential distal metabolic targets, enzymes of glucose and fatty acid metabolism and oxidative phosphorylation in the rat visceral fat depot, that is, retroperitoneal WAT (RpWAT) during cold acclimation.

## 2. Material and Methods

### 2.1. Animals and Experimental Protocol

Three-month-old male Mill Hill hybrid hooded rats (*Rattus norvegicus, Berkenhout 1769*) were housed under a 12/12 h reverse light/dark cycle and fed standard chow and water *ad libitum*. The animals were divided into two main groups: one group was kept at room temperature (22 ± 1°C) for the entire experimental period and acted as the control, and the other group was kept in the cold (4 ± 1°C). The duration of cold exposure ranged from 1 to 45 days (1, 3, 7, 12, 21, and 45 days), with six animals per experimental group. 

The experiments were approved by the Ethical Committee for the Treatment of Experimental Animals of the Institute for Biological Research “Siniša Stanković,” Belgrade.

### 2.2. Sample Collection and Storage

At the end of the experimental periods (after day 1, 3, 7, 12, 21 or 45 of cold acclimation), the rats were fasted overnight and killed by decapitation. RpWAT depot (from the left side) was quickly dissected free of vessels and connective tissue and placed on ice. These tissues were then snap-frozen in liquid nitrogen, and stored at −80°C until subsequent analysis.

For western blot analysis, a portion of RpWAT, weighing 400 mg obtained from each rat was homogenized (Ultra/Turrax homogenizer, Janke und Kunkel Ka/Werke, Staufen, Germany, 0–4°C) in 0.25 M sucrose, 0.1 mM EDTA and 50 mM Tris-HCl buffer, pH 7.4, containing 10 *μ*g mL^−1^ protease inhibitor cocktail (Roche Diagnostic GmbH, Mannheim, Germany). Homogenates were sonicated for 10 s at 40 kHz and centrifuged at 38 000 g for 90 min [30].

Protein concentration in the supernatant was estimated by the method of Lowry et al. [31] using bovine serum albumin as a standard. Extracts were stored at −80°C until western blot analysis.

Proteins were resolved according to Laemmli [32]. Ten micrograms of protein aliquots was boiled, electrophoresed in SDS-polyacrylamide gel, and transferred to Hybond-P polyvinylidene fluoride membranes (Amersham, Piscataway, NJ, USA). Nonspecific binding sites of the membranes were blocked by 5% BSA in TBS (200 mM Tris, 1.5 M NaCl, pH 7.4) for 1 h at room temperature. The blots were then incubated with a specific primary antibody in TBS-T (0.2% Tween-20 and 5% BSA in TBS) against adiponectin (1 : 5000), resistin (1 : 2000), catalytic *α* subunit phosphorylated on Thr172 of AMPK (2 *μ*g mL^−1^), purchased from Millipore Corporation, Temecula, CA, USA, and HIF-1*α* (1 : 2000), glyceraldehyde-3-phosphate dehydrogenase (GAPDH; 1 : 1000), the E1 beta subunit of pyruvate dehydrogenase (PDH; 1 *μ*g mL^−1^), medium chain fatty acids acyl-CoA dehydrogenase (ACADM; 1 : 10000), acetyl-CoA carboxylase (ACC; 1 : 1000), the subunit IV of cytochrome *c* oxidase (COX IV; 0.1 *μ*g mL^−1^), ATP synthase (0.8 *μ*g mL^−1^) and against *β* actin (1 : 1000), purchased from Abcam (Cambridge, UK). Incubation was carried out overnight at 4°C followed by a 2 h incubation period at room temperature with a horseradish peroxidase-conjugated IgG secondary antibody. Goat anti-mouse secondary antibody (Santa Cruz Biotechnology, CA, USA) was used for detection of resistin, GAPDH, cytochrome *c* oxidase, ATP synthase, and *β* actin, while goat anti-rabbit secondary antibody (Santa Cruz Biotechnology) was used for detection of adiponectin, phospho-AMPK*α*, HIF-1*α*, PDH, ACADM, and ACC, respectively. The protein bands were visualized by chemiluminescence using the chemiluminescence detection system from Amersham (API, Indianapolis, IN, USA). The intensity of the bands was quantified with ImageQuant software. Volume was the sum of all the pixel intensities within a band; that is, 1 pixel = 0.007744 mm^2^. We averaged the ratio of dots per band for the target protein and *β* actin in corresponding time-periods, from three similar independent experiments, and expressed them relative to the room temperature-acclimated control, which was standardized as 100%. The data were then analyzed statistically.

### 2.3. Statistical Analysis

Analysis of variance (ANOVA) followed by Tukey's post hoc test was used to calculate statistical differences using GraphPad Prism statistical software (Version 3.03, San Diego, CA, USA). All results are expressed as means ± S.E.M. with the significance threshold set at *P* < 0.05.

## 3. Results

### 3.1. Protein Contents of Adiponectin and Resistin

**Figure 1:**
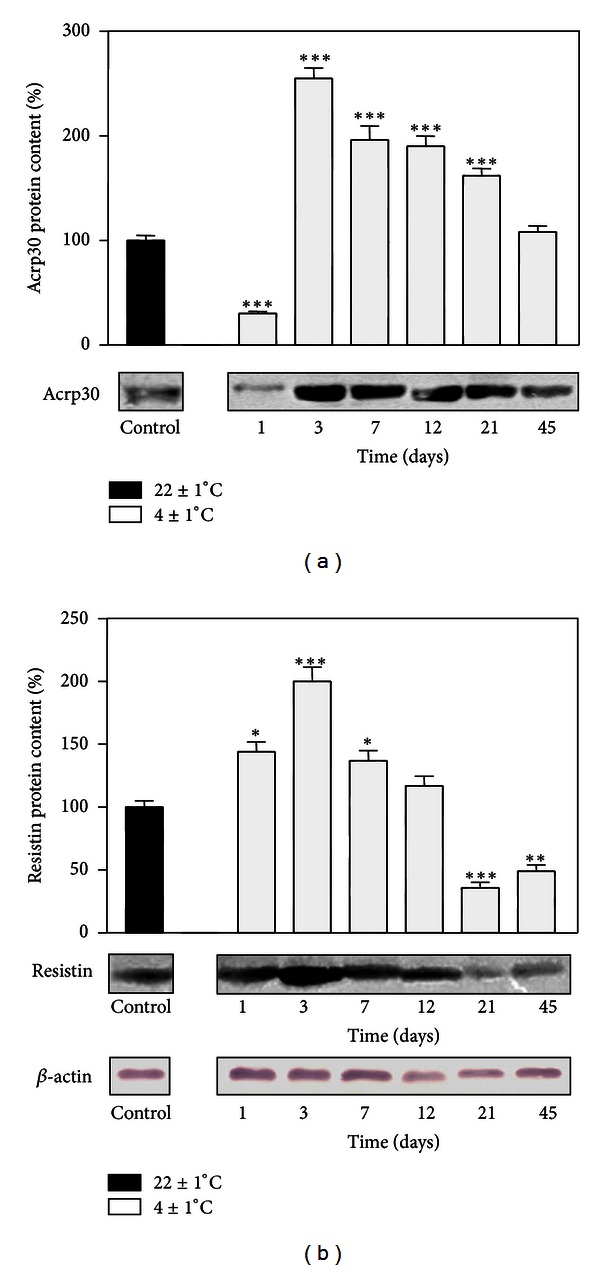
Time course of the changes in protein expression of adiponectin (Acrp30) (a) and resistin (b) in rat RpWAT during cold acclimation. Protein content is expressed relative to a control acclimated to room temperature, which was standardized as 100%. The results of the representative example from three observations are shown. Data were quantified as described in Section 2. The values represent the mean ± S.E.M. *Compared to control, *P < 0.05, **P < 0.01, and ***P < 0.001.

Figure 1 shows time-dependent changes in the levels of adiponectin and resistin in RpWAT during cold acclimation. Compared to the control, the protein level of adiponectin (Figure 1(a)) markedly decreased after day 1 of cold exposure (*P* < 0.001) but then sharply increased from day 3 to day 21 of cold exposure (*P* < 0.001).

The results of western blotting showed that the resistin protein level, compared to the control, increased on days 1 (*P* < 0.05), 3 (*P* < 0.001), and 7 (*P* < 0.05) and, thereafter, on days 21 (*P* < 0.001) and 45 (*P* < 0.01) significantly decreased (Figure 1(b)).

### 3.2. Changes in HIF-1*α* and Phospho-AMPK*α* Expression

**Figure 2:**
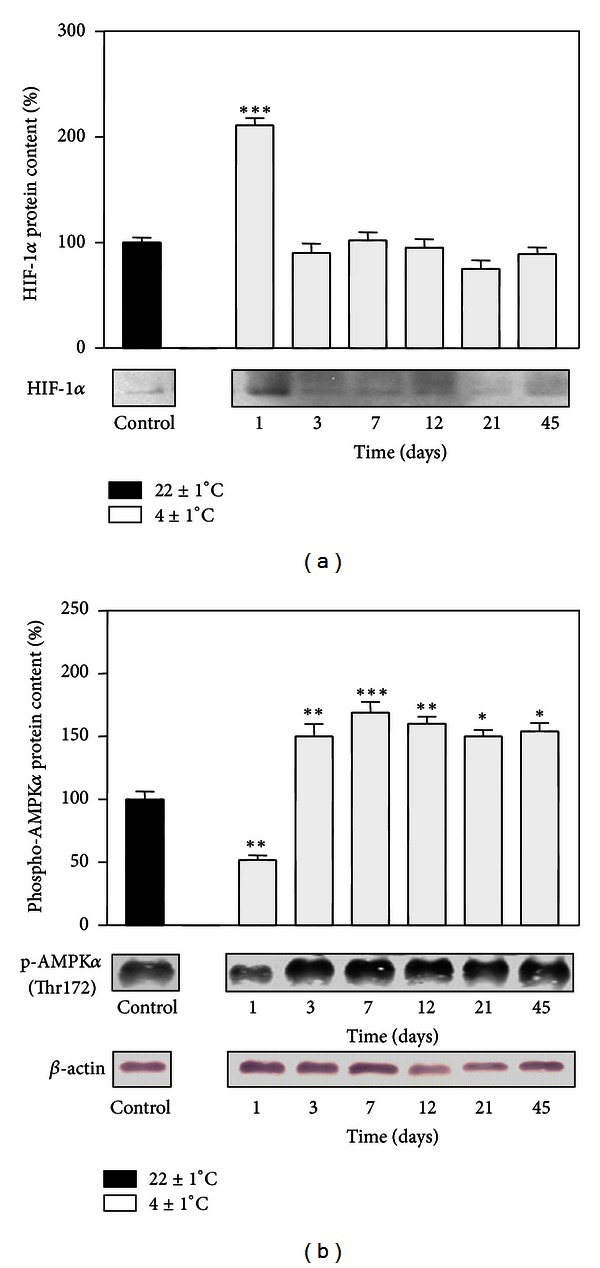
Protein levels of HIF-1*α* (a) and phospho-AMPK*α* (b) in rat RpWAT during cold acclimation. Protein content is expressed relative to a room temperature-acclimated control, which was standardized as 100%. The results of the representative example from three observations are shown. Data were quantified as described in Section 2. The values represent the mean ± S.E.M. *Compared to control, *P < 0.05, **P < 0.01, and ***P < 0.001.

As shown in Figure 2(a), HIF-1*α* protein expression level was markedly induced on the first day of cold exposure (*P* < 0.001) but returned to the control level after 3 days and this level was maintained throughout the entire acclimation period. By contrast, on day 1 of cold exposure, phospho-AMPK*α* protein level significantly decreased (*P* < 0.01). However, starting from day 3 until the end of the cold acclimation period, phospho-AMPK*α* protein level was notably increased compared to the control (Figure 2(b)). 

### 3.3. Protein Expression of Key Enzymes of Glucose (GAPDH and PDH) and Lipid (ACADM and ACC) Metabolism and Proteins Mediating Oxidative Phosphorylation (Cytochrome *c*  Oxidase and ATP Synthase)

**Figure 3:**
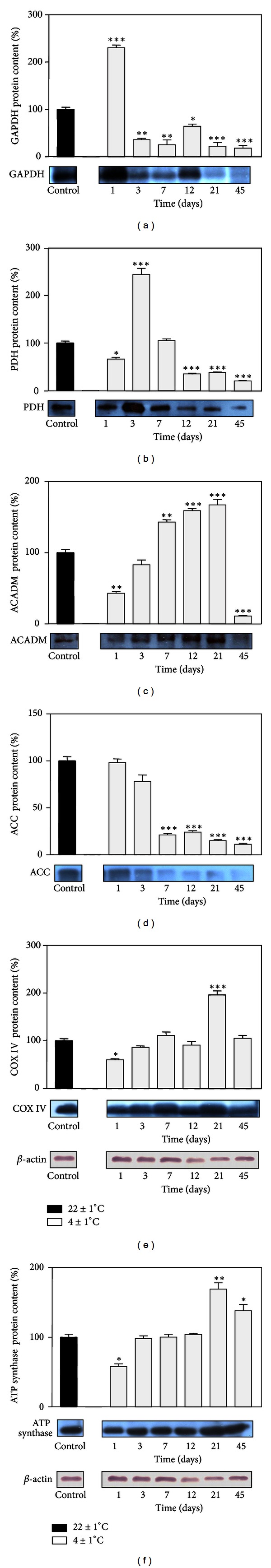
Changes in GAPDH (a), PDH (b), ACADM (c), ACC (d), subunit IV of cytochrome *c*  oxidase (COX IV) (e), and ATP synthase (f) protein levels in rat retroperitoneal depot of white fat tissue after different time periods of cold exposure. Protein content is expressed relative to the control maintained at room temperature, which was standardized as 100%. The results of the representative example from three observations are shown. Data were quantified as described in Section 2. The values represent the mean ± S.E.M. *Compared to control, *P < 0.05, **P < 0.01, and ***P < 0.001.

Of the many proteins involved in the metabolic response, we examined GAPDH, PDH, ACADM, ACC, cytochrome *c* oxidase, and ATP synthase. The level of the glycolytic enzyme, GAPDH (Figure 3(a)), markedly increased on day 1 of cold exposure but significantly decreased on prolonged cold acclimation, starting from day 3. On the other hand, protein levels of PDH, the enzyme that regulates the flow of energy in cells by determining when pyruvate should be used for oxidative phosphorylation versus “neutralized” to lactic acid, declined after day 1 (*P* < 0.05) but sharply increased on day 3 (*P* < 0.001) of cold exposure (Figure 3(b)). However, the protein level of PDH significantly decreased on prolonged cold acclimation, that is, after 12 days of cold acclimation. 

Figures 3(c) and 3(d) show the time-dependent changes in the level of enzymes involved in fatty acid metabolism (oxidation and biosynthesis), ACADM and ACC, respectively. The ACADM protein level, after an initial decrease observed after day 1 (*P* < 0.01), increased on days 7 (*P* < 0.01), 12 and 21 (*P* < 0.001) reaching statistical significance. However, during late cold acclimation (after 45 days) the ACADM protein level decreased below the control level (*P* < 0.001). At the same time, the level of ACC, the enzyme that controls fatty acid biosynthesis and inhibits fatty acid oxidation, significantly decreased (*P* < 0.001) on days 7, 12, 21 and 45 of cold acclimation. 

Figures 3(e) and 3(f) shows that the protein contents of both examined components of oxidative phosphorylation, cytochrome *c* oxidase and ATP synthase, respectively, decreased below the control level (*P* < 0.05) on day 1 of cold exposure. However, after day 3 of cold exposure, both these proteins returned to control values and even increased above the controls; cytochrome *c* oxidase increased after days 21 (*P* < 0.001) and ATP synthase after day 21 (*P* < 0.01) and 45 (*P* < 0.05) of cold acclimation.

## 4. Discussion

The time-dependent expression changes in adiponectin and resistin in RpWAT during cold acclimation show that these factors are important regulators that integrate white fat metabolic response and the roles of this tissue plays in adaptation to cold. The observed inverse expression profile of HIF-1*α* and phospho-AMPK*α* on cold exposure strongly suggests that the interplay between these molecules integrates adiponectin level and metabolic remodeling of RpWAT from dormant tissue to tissue with higher oxidative capacity. Namely, on day 1 of cold exposure, the HIF-1*α* protein level increased in parallel with a decrease in the level of adiponectin, phospho-AMPK*α*, and important enzymes indicative of oxidative metabolism (PDH, ACADM, ATP synthase, and cytochrome *c* oxidase), while the reverse was seen after 1 day of cold exposure; the normalization of HIF-1*α* was coupled with an increase in adiponectin, activation of AMPK*α*, and all the above-mentioned metabolic enzymes. Resistin protein level increased during early cold acclimation (first 7 days), and its decrease following thermogenic adaptation (after 21 days) is in accordance with its role in the regulation of overall fatty acids availability. RpWAT endocrine and metabolic remodeling during cold acclimation is discussed in more detail below.

Adiponectin (also known as apM1, AdipoQ, Gbp28, and Acrp30) is a basic adipokine involved in energy homeostasis. It acts on the brain by stimulating centers for food intake and on the periphery by stimulating glucose utilization and fatty acid oxidation [19, 33] which are increased on cold exposure as a part of the physiological adaptation to low temperature.

However, data regarding the *in vivo* energy metabolism in white fat tissue especially in relation to adiponectin synthesis and its effects are limited. In this study, we observed that adiponectin protein level decreased after one day of cold exposure. This result is in agreement with the data showing that conditions associated with acute beta adrenergic stimulation reduces serum adiponectin and its mRNA level in WAT [34–36]. The main molecular mediator of adiponectin acting in all target tissues is AMPK*α* [19, 20, 37–39]. Acting through AMPK*α*, adiponectin regulates a number of proteins involved in glucose uptake and energy metabolism to increase catabolic pathways, that is, to switch off anabolic pathways that consume ATP [22]. Accordingly, we found that in parallel with the decrease in adiponectin on day 1 of cold exposure there was a decrease in the protein level of phosho-AMPK*α* as well as the enzymes involved in oxidative metabolism, PDH, ACADM, ATP synthase, and cytochrome *c* oxidase. Simultaneously, the protein levels of HIF-1*α* and GAPDH increased, suggesting that, at the initial stage of cold exposure, WAT relies on the glycolytic strategy for energy production. Such metabolic pathway preference for glucose in RpWAT after only one day of cold exposure is in line with the low capacity of white fat tissue for fatty acid oxidation and its role in providing fatty acids, the main energy fuel for thermogenic effector tissues during cold acclimation.

However, these changes in the above-mentioned molecules were reversed after 3 days of cold exposure: HIF-1*α* and GAPDH decreased compared to the control, while increase in adiponectin paralleled the activation of AMPK*α*. This was followed by a sequential restoration/increase in the enzymes involved in oxidative metabolism (PDH, ACADM, ATP synthase, and cytochrome *c*  oxidase). Also, these changes parallel by decrease in relative RpWAT mass and are in agreement with temporal changes in most antioxidative enzyme activities in this depot which also increased significantly, as we found previously in RpWAT, after 3 days of cold acclimation [40]. We hypothesize that these enzymes increased as a compensatory response following the intensified production of reactive oxygen species due to increased oxidative metabolism. The results of the present study support this hypothesis. According to all, it is suggested that oxidative metabolism increases in RpWAT during prolonged cold exposure and that increased adiponectin/AMPK*α* signaling mediates WAT metabolic remodeling toward lipid oxidation.

In accordance, we also observed a progressive decrease in the lipogenic enzyme, ACC, from day 7 of cold exposure. ACC synthesizes malonyl-CoA from acetyl-CoA [41]. Decreased malonyl-CoA level, resulting from decreased ACC protein level is a known stimulus for increased *β*-oxidation capacity, because it reduces the entry of fatty acids into mitochondria [22]. These findings support a gradual increase in the key *β*-oxidation enzyme, ACADM, which reached statistical significance on day 7 of cold exposure. From these results, it seems likely that during sustained cold exposure this tissue oxidizes fatty acids for its energy needs. Also, it is known that ACC is one of the main downstream target proteins for AMPK*α* phosphorylation, that is, adiponectin signaling [22, 42], so thus here observed decrease of ACC protein level, in parallel with phospho-AMPK*α* and adiponectin level increase, in RpWAT on sustained cold exposure, indicate that adiponectin/AMPK*α* signaling restrains lipogenic pathways and alleviates the inhibition for fatty acid oxidation in RpWAT on sustained cold by decreasing ACC content.

Molecular changes of RpWAT toward a tissue with higher oxidative capacity, presented in this study, are a reflexion of the complex functional (metabolic and endocrine) adaptations and structural remodeling of the tissue. This structural aspects in some degree may involve the emergence of brown fat like cells. Namely, it is evidenced that chronic stimulation of beta 3-adrenergic signaling, including the cold exposure promotes the expression of brown fat cell characteristic genes, such as uncoupling protein 1 [43, 44], and causes the emergence of brown fat-like cells [10, 45, 46], in adipose tissues conventionally considered as white fat. Thus, it would be interesting to define links between the here presented metabolic remodeling of RpWAT and the eventual alterations in the cellular composition within this depot.

The expression pattern of resistin in RpWAT is consistent with the described changes in the metabolic behavior of this tissue during cold acclimation. It is known that resistin interferes with lipid metabolism in adipocytes, primarily by inhibiting fatty acid reesterification and increasing their release from adipocytes [23]. Thus, resistin expression was increased early during cold exposure (in the first 7 days), when the RpWAT metabolic program was organized to supply other tissues with fatty acids and decreased in the late periods of cold acclimation (after 21 days) when thermogenic homeostasis was achieved. The expression and role of resistin were examined mostly from the pathophysiological aspect. However, results obtained in this study provide new insights into the physiological significance of resistin as an important regulator of fatty acid reesterification, that is, fatty acid mobilization from WAT.

## 5. Conclusions

In summary, we showed that adiponectin is an important endocrine regulator in metabolic adaptation to cold acclimation. More importantly, adiponectin and resistin finely adjust the metabolic response of white fat tissue itself. This metabolic remodeling initially involves fatty acid mobilization on cold exposure and subsequent oxidation combustion during cold acclimation. Our results strongly suggest that AMPK*α* integrates metabolic and endocrine aspects of white fat metabolic profile changes on cold exposure. Withal, here presented study indicates important molecules, acting in the white fat tissue conversion toward a tissue with higher oxidative capacity that may help in the design of effective therapies for obesity.
